# Unraveling GDAP1: Bridging Mitochondrial Biology and Peripheral Neuropathy

**DOI:** 10.3390/biom16020280

**Published:** 2026-02-10

**Authors:** Lara Cantarero, Janet Hoenicka, Francesc Palau

**Affiliations:** 1 Laboratory of Neurogenetics and Molecular Medicine, Center for Genomic Sciences in Medicine, Institut de Recerca Sant Joan de Déu, 08950 Barcelona, Spain; 2 Centro de Investigación Biomédica en Red de Enfermedades Raras (CIBERER), ISCIII, 08950 Barcelona, Spain; 3 Hospital Sant Joan de Déu, 08950 Barcelona, Spain; 4 Division of Pediatrics, Faculty of Medicine and Health Sciences, University of Barcelona, 08007 Barcelona, Spain

**Keywords:** axonopathy, Charcot-Marie-Tooth disease, GDAP1, lysosomes, membrane contact sites, mitochondria, peroxisomes, neuron, axon, glial cells, outer mitochondrial membrane, neuroinflammation, neuropathies

## Abstract

The mitochondrial outer membrane (OMM) plays a crucial role in maintaining cellular homeostasis by regulating mitochondrial dynamics, organelle interactions, and stress responses. In peripheral neurons—cells with high metabolic demands and long axons—the OMM acts as a vital platform for coordinating bioenergetics, calcium signaling, and redox balance. Ganglioside-induced differentiation-associated protein 1 (GDAP1), an OMM-anchored protein, has emerged as a key regulator of mitochondrial fission and transport, redox homeostasis, and mitochondrial membrane contact sites (MCSs). Genetic variants in GDAP1 cause Charcot–Marie–Tooth disease (CMT), emphasizing its essential role in peripheral nerve function. This review highlights the multifaceted functions of GDAP1 in neuronal physiology and as a model protein that integrates organelle communication and mitochondrial biology. We further discuss how GDAP1 dysfunction leads to structural and functional impairments in peripheral neurons, proposing the OMM and its microenvironment as critical targets for therapeutic intervention in inherited neuropathies.

## 1. Introduction

Mitochondria are highly dynamic and compartmentalized organelles that serve as central hubs of cellular metabolism, energy production, calcium homeostasis, and apoptotic signaling [1,2,3]. Among the proteins associated with mitochondrial function, Ganglioside-induced differentiation-associated protein 1 (GDAP1) has emerged as a key regulator of neuronal mitochondrial homeostasis. GDAP1 is an outer mitochondrial membrane (OMM)-anchored protein predominantly expressed in neurons [4], whose genetic variants cause Charcot–Marie–Tooth disease (CMT) [5,6], a group of inherited peripheral neuropathies characterized by distal axonal degeneration and progressive muscle weakness. Despite its clear clinical relevance, the precise molecular mechanisms through which GDAP1 contributes to mitochondrial function and neuronal integrity remain incompletely understood.

Structurally, mitochondria are defined by two membranes: the inner mitochondrial membrane (IMM), which encloses the matrix and cristae and contains the electron transport chain, and the outer mitochondrial membrane, which serves as the interface between mitochondria and the rest of the cell [7]. While the IMM and matrix have long been the main focus of mitochondrial biology, the OMM is increasingly recognized as a critical platform for integrating cellular signals and coordinating communication between organelles [2]. This emerging view is particularly relevant for GDAP1, given its localization to the OMM and its involvement in multiple processes that require dynamic inter-organelle coordination.

**Figure 1:**
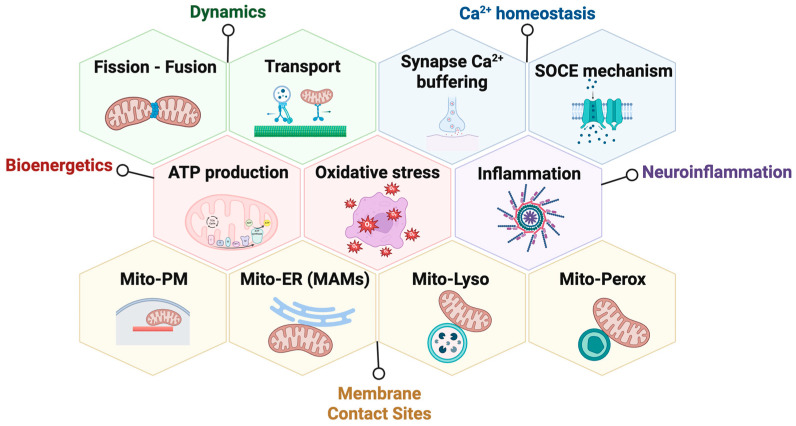
The mitochondrial outer membrane protein GDAP1 serves as a central hub for various cellular functions. GDAP1 is involved in mitochondrial dynamics, calcium homeostasis, mitochondrial bioenergetics, neuroinflammation, and mitochondrial membrane contact sites. Abbreviations: SOCE: store-operated calcium entry; Mito: mitochondria; PM: plasma membrane; ER: endoplasmic reticulum; MAMs: mitochondria-associated membranes; Lyso: lysosome; and Perox: peroxisome. Illustration created with BioRender. Lara Cantarero (2026). https://www.biorender.com/.

The OMM hosts several proteins involved in mitochondrial network dynamics, protein import, apoptosis, and the formation of mitochondrial membrane contact sites (MCSs) with other organelles such as the endoplasmic reticulum (ER), the plasma membrane (PM), peroxisomes, lysosomes, and lipid droplets [8,9]. These MCSs are essential for exchanging calcium, lipids, and metabolites, as well as coordinating adaptive responses to cellular stress. Disruption of these contact sites is now understood to contribute to the pathophysiology of various diseases, including neurodegenerative disorders [10,11]. In this context, GDAP1 has been linked to mitochondrial fission and axonal transport, bioenergetics, redox sensing, calcium signaling, neuroinflammation, and the regulation of MCSs [12,13,14,15] (Figure 1), positioning it as a multifunctional regulator at the crossroads of mitochondrial dynamics and inter-organelle communication.

This review highlights the emerging role of the OMM as a dynamic and integrative platform in neuronal cells, using GDAP1 as a model protein to examine the mechanisms linking mitochondrial function, organelle communication, and peripheral nerve pathology. We focus on the physiological roles of GDAP1 and the consequences of its dysfunction, positioning the OMM as a key site of vulnerability—and a potential therapeutic target—in peripheral neuropathies.

## 2. Discovery, Evolution, and Expression of the *GDAP1* Gene and Protein

*GDAP1* was initially identified as one of ten genes upregulated in the Neuro2a mouse neuroblastoma cell line when differentiated by overexpression of GD3 ganglioside synthase [16]. These cDNAs were collectively called GDAP1 to GDAP10, referencing ganglioside-induced differentiation-associated proteins. This discovery positioned GDAP1 as a potential regulator of neuronal differentiation and prompted subsequent studies on its molecular and cellular functions.

Orthologues of *GDAP1* have been identified across a broad range of vertebrate species, from mammals to fish, highlighting its evolutionary conservation. Notably, GDAP1 paralogs—most notably the closely related human gene *Ganglioside induced differentiation associated protein 1 like 1 (GDAP1L1)*—are also found in various vertebrates. Beyond vertebrates, GDAP1-related sequences with conserved domains and predicted structural homology have been observed in invertebrates. Remarkably, *GDAP1*-like genes have been detected in certain plant species and, unexpectedly, in bacterial genomes, suggesting an ancient evolutionary origin. Comparative genomic analyses propose that GDAP1-like proteins emerged early in eukaryotic evolution, before the divergence of plant and animal lineages [17].

In humans, the *GDAP1* gene is located on chromosome 8q21.11 [5,18]. It spans approximately 17 kb and comprises six exons and five introns. *GDAP1* is primarily expressed in the nervous system, particularly in neurons [4,6], but is also detected in Schwann cells [14]. Neuronal localization studies have revealed GDAP1 expression in motor neurons of the spinal cord, sensory neurons of the dorsal root ganglia, Purkinje neurons of the cerebellum, pyramidal neurons of the cortex, and mitral cells of the olfactory bulb [19]. Additionally, *GDAP1* expression is developmentally regulated: it becomes detectable at embryonic stage E13, gradually increases during development, and peaks in adulthood [16], highlighting its essential role in neuronal maturation and maintenance.

### GDAP1 Is an Atypical Glutathione S-Transferase Protein

The *GDAP1* gene encodes the GDAP1 protein, which phylogenetic and structural analyses classify as a member of a distinct subfamily within the glutathione S-transferase (GST) superfamily [6,17]. GDAP1 has three main regions: (1) two canonical N-terminal GST domains, which, despite lacking classical enzymatic activity, are thought to mediate redox-related signaling [20,21]; (2) an α4–5 loop region involved in protein–protein interactions and structural stabilization; and (3) two hydrophobic C-terminal domains that form a transmembrane region anchoring the protein to the OMM [22]. Recently, the first full-length structural model of human GDAP1 has been reported [23]. Using synchrotron-based approaches, the authors showed that GDAP1 interacts with the membrane through a single transmembrane helix flanked by two peripheral helices that engage with the outer and inner leaflets of the OMM [23]. This arrangement positions the N-terminal GST domains toward the cytosol, where GDAP1 may interact with cytosolic partners or signaling complexes [22].

Although GDAP1 has two GST-like domains, its inherent catalytic activity remains debated. Several studies have not detected glutathione (GSH) binding or classical GST activity [21], while others have reported GST-like activity under specific expression conditions [20]. Recent structural analyses have helped resolve this inconsistency by showing that GDAP1 cannot bind GSH at the canonical G-site, although it can still bind substrates at the H-site [24,25]. These findings suggest that GDAP1 may not function as a conventional GST enzyme but could instead exert a non-canonical, context-dependent activity, potentially related to redox-sensitive substrate binding or membrane-associated processes. Alternatively, GDAP1 may primarily act as a regulatory protein, modulating redox balance and organelle communication through protein–protein interactions rather than through direct catalytic activity. At present, the relative contribution of these potential mechanisms remains unresolved, highlighting the need for further biochemical and cellular studies.

## 3. *GDAP1* Clinical Variants and Disease

Clinical variants in the *GDAP1* gene cause Charcot–Marie–Tooth disease, the most common hereditary motor and sensory neuropathy. *GDAP1*-related CMT give rise to a broad phenotypic spectrum that includes several clinical subtypes, most of which are axonal neuropathies, inherited either in a recessive [5,6,26] or dominant manner [27,28,29]. Autosomal recessive variants (classified as CMT4A) are usually linked to severe, early-onset neuropathy, often appearing during infancy or early childhood. Affected individuals frequently experience rapid disease progression, often resulting in wheelchair dependence by the second decade of life, and vocal cord paresis is commonly observed as a characteristic feature [30,31]. Conversely, autosomal dominant mutations (classified as CMT2K) generally present a milder, later-onset phenotype, characterized by slowly progressing distal weakness and sensory loss, with maintained ambulation into adulthood [32].

**Figure 2:**
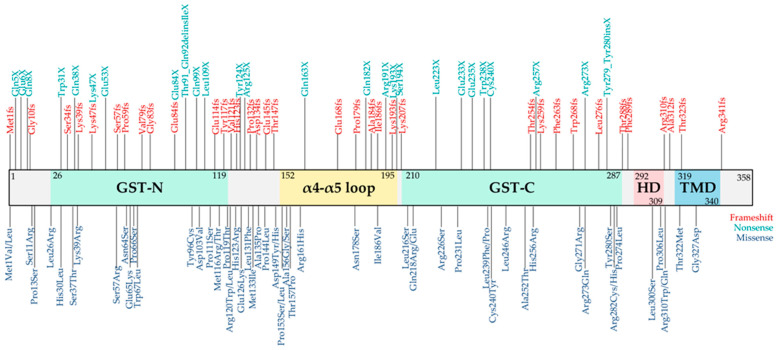
Schematic illustration of the GDAP1 protein domains highlighting the most significant clinical variants. Glutathione-S transferase (GST), hydrophobic domain (HD), transmembrane domain (TMD), and amino acid residues numbering are shown. The number indicates the amino acid position in GDAP1 protein. Clinical variants reported in ClinVar (last access 30 October 2025) were represented: frameshift (red), nonsense (green), and missense (blue).

To date, over 130 pathogenic *GDAP1* variants have been reported across diverse populations (Figure 2), including missense, nonsense, and frameshift variants. Their distribution throughout the *GDAP1* coding sequence highlights the importance of specific protein domains. The pathogenic effect of each variant depends not only on its location within these functional domains but also on the type of amino acid substitution and its impact on protein stability, folding, or mitochondrial localization. Collectively, the molecular diversity of *GDAP1* variants reflects the clinical heterogeneity seen in CMT patients, emphasizing the need to integrate genetic, structural, and functional data to better understand disease mechanisms and support emerging precision medicine approaches.

Additionally, digenic inheritance involving *GDAP1* and *MFN2* (which encodes Mitofusin 2) has become an important mechanism in CMT disease [33,34,35], highlighting the complex genetic basis of inherited neuropathies. Therefore, having variants in both genes may produce a more severe or unusual phenotype, indicating synergistic effects on mitochondrial morphology, axonal transport, and neuronal survival [36]. The clinical spectrum includes early-onset, progressive distal weakness and sensory loss, often with pyramidal signs or optic atrophy. The interaction between GDAP1 and MFN2 [37] and their shared location in the OMM underscore the fundamental role of the mitochondrial membranes in maintaining the health of peripheral nerves [38] and would explain how digenic inheritance contributes to phenotypic variability in CMT.

## 4. GDAP1: A Multifunctional OMM Protein

This section reviews the molecular properties and physiological functions of GDAP1 that support its role in maintaining peripheral nerve homeostasis, as well as the pathological effects of *GDAP1* clinical variants on these cellular processes.

### 4.1. Mitochondrial Dynamics: From Fission–Fusion Balance to Axonal Transport

Mitochondrial dynamics refer to the continuous remodeling of mitochondrial architecture governed by the antagonistic yet coordinated processes of fusion and fission, as well as the axonal transport of mitochondria to regions of high energy demand. These dynamic processes enable mitochondria to adapt their morphology and distribution in response to cellular metabolic demands and environmental cues.

#### 4.1.1. GDAP1 as a Non-Canonical Mitochondrial Fission Factor

**Figure 3:**
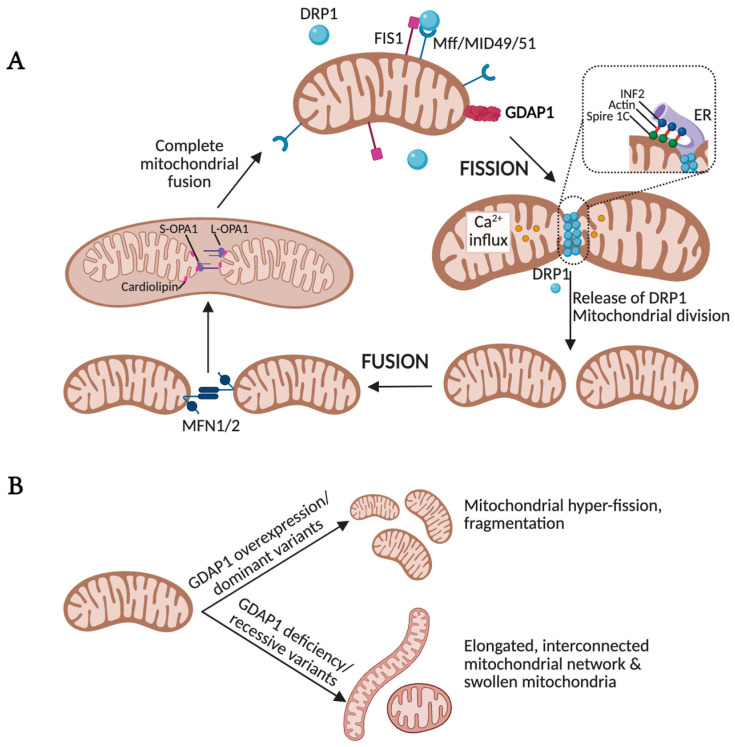
GDAP1 is involved in mitochondrial network dynamics. (**A**) Fission and fusion processes regulate mitochondrial morphology and, in extension, its function, allowing the mitochondrial network to adapt to the needs of cells. FIS1 and DRP1, which oligomerize, induce GTP hydrolysis-mediated membrane constriction for mitochondrial fission. The activity of DRP1 is supported by actin polymerization at the ER-mitochondria interface mediated by the actin-nucleating proteins inverted formin 2 (INF2) and formin-binding protein Spire 1C. This actin filament accumulation could drive initial mitochondrial constriction that supports subsequent DRP1 polymerization. Fusion relies on two GTPases residing at both the OMM and the IMM, Mitofusin and OPA1, respectively. (**B**) GDAP1 overexpression or dominant GDAP1 variants promote mitochondrial hyper-fission, whereas GDAP1 deficiency or recessive variants result in elongated, interconnected mitochondrial networks and swollen mitochondria with disorganized cristae. Illustration created with BioRender. Lara Cantarero (2026). https://www.biorender.com/.

Mitochondria form highly dynamic tubular and branched networks that are continuously remodeled through tightly regulated fusion and fission events (Figure 3) [39]. This structural plasticity allows cells to adapt mitochondrial function to changing physiological and metabolic needs. Disruption of the balance between fusion and fission is associated with a wide range of genetic and neurodegenerative disorders, underscoring the central role of mitochondrial dynamics in maintaining cellular health [37,40,41,42,43]. The molecular machinery that governs these processes is extensively regulated by post-translational modifications, which couple mitochondrial morphology to intracellular signaling pathways and help fine-tune mitochondrial responses based on the cell’s functional state [44].

In mammalian cells, mitochondrial fusion is mediated by two OMM-anchored GTPases, Mitofusin 1 (MFN1) and MFN2, both members of the dynamin-related protein family. Fusion of the inner mitochondrial membrane (IMM) is coordinated by OPA1, another dynamin-related GTPase located in the IMM and intermembrane space [45]. In contrast, mitochondrial fission is primarily driven by Dynamin-related protein 1 (DRP1), recruited from the cytosol to the OMM. There, DRP1 interacts with several receptor proteins, including mitochondrial fission factor (MFF), mitochondrial dynamics proteins of 49 and 51 kDa (MID49, MID51), and mitochondrial fission 1 protein (FIS1). Upon binding, DRP1 oligomerizes into helical structures that constrict and ultimately divide the mitochondrial membrane [46]. Failure to maintain the dynamic balance between fusion and fission results in either mitochondrial fragmentation or hyperfusion, both of which disrupt axonal transport, compromise synaptic function, and promote cell death—common hallmarks of neurodegenerative and neuromuscular diseases.

GDAP1 has emerged as a non-canonical mitochondrial fission factor, as no homologs have been identified in *Caenorhabditis elegans* or *Saccharomyces cerevisiae* [17]. Its fission-promoting activity requires canonical components of the fission machinery, including DRP1 and FIS1 [47] and it has been proposed that the fission activity of GDAP1 is regulated by its C-terminal HD1 domain. Through the amphipathic properties of HD1, GDAP1 promotes membrane curvature, thereby driving membrane fission [20]. Interestingly, human GDAP1 rescues the abnormally large phenotype caused by Fis1 deficiency in *S. cerevisiae* [48].

Overexpression of GDAP1 drives mitochondrial fragmentation [14], whereas GDAP1 deficiency results in elongated, interconnected mitochondrial networks (Figure 3) [14,49,50,51]. High-resolution analyses of GDAP1-deficient cells have revealed two distinct pathological mitochondrial phenotypes: (i) elongated, hyperfused mitochondria, and (ii) spheroidal, swollen organelles with disorganized cristae [12,13]. Quantitative network profiling further demonstrates increased mitochondrial volume and length, accompanied by a higher number of branchpoints and endpoints. Conversely, the number of discrete mitochondrial units is markedly reduced, and network distribution within the cell becomes abnormal. Collectively, these findings indicate that GDAP1 loss impairs mitochondrial fission, leading to disorganization of both membrane architecture and network topology. Remarkably, the phenotypic consequences of GDAP1 clinical variants appear to depend on the mode of inheritance. Recessive variants are typically associated with reduced fission activity, consistent with a loss-of-function mechanism, whereas dominant mutations enhance mitochondrial fragmentation, suggesting a gain-of-function effect [47,52]. This dual pathogenic behavior underscores the mechanistic complexity of GDAP1 and highlights its central role in maintaining mitochondrial architecture and its contribution to disease pathogenesis.

#### 4.1.2. The Role of GDAP1 in Mitochondrial Transport Along Axons

**Figure 4:**
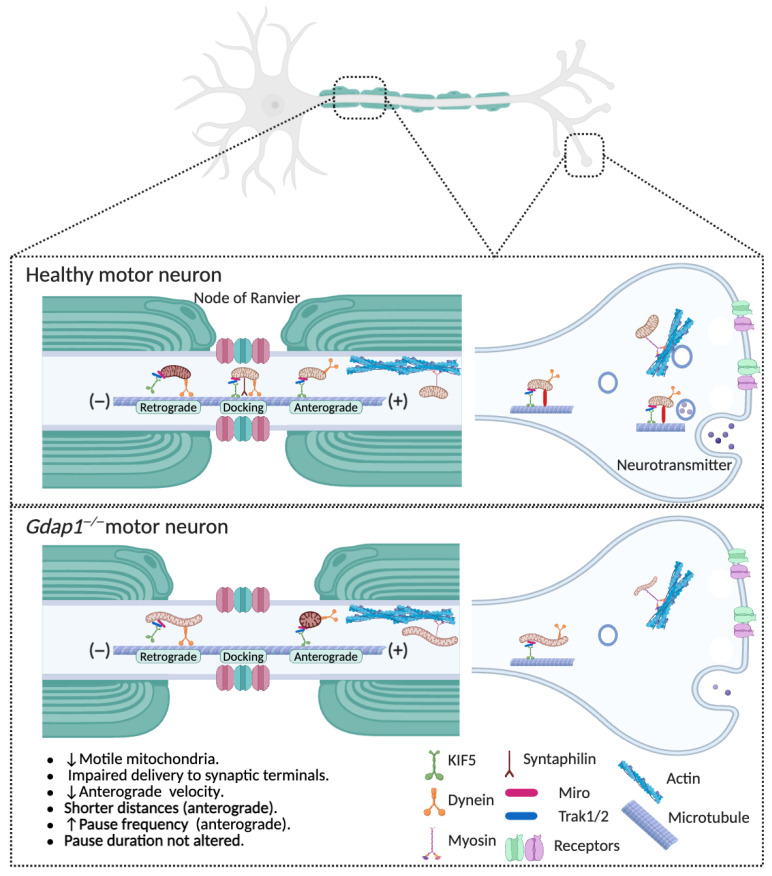
GDAP1 deficiency impairs mitochondrial axonal transport. Upper panel: The movement of mitochondria from the soma to distal axonal and dendritic regions depends on the polarity and organization of neuronal microtubules. In axons, cytoplasmic dynein drives retrograde transport of mitochondria back to the soma, while kinesin motors mediate anterograde transport toward distal axonal regions and synaptic terminals. In dendrites, where microtubules exhibit mixed polarity, both kinesin and dynein motors transport mitochondria bidirectionally. Myosin motors likely facilitate short-range mitochondrial movements within presynaptic terminals, growth cones, and dendritic spines, where actin filaments form the main cytoskeletal scaffold. Mobile mitochondria can become anchored to stationary pools through interactions between the docking receptor syntaphilin and microtubules. These anchoring mechanisms ensure proper spatial distribution of stationary mitochondria within axons and synapses. Lower panel: Loss of GDAP1 severely disrupts mitochondrial trafficking in neurons, reducing the number and mobility of mitochondria. Both anterograde transport to distal regions and retrograde return to the soma are impaired, leading to abnormal mitochondrial distribution, energy deficits, and neuronal degeneration. As a result, these defects decrease the delivery of healthy mitochondria to distal regions and hinder the clearance of damaged ones, ultimately disrupting synaptic function. Illustration created with BioRender. Lara Cantarero (2026). https://www.biorender.com/.

Mitochondrial trafficking is essential for all cell types because it ensures mitochondria are delivered to parts of the cell where energy production and calcium buffering are required. In neurons, this process is fundamental due to the great distances between the soma—where mitochondrial biogenesis occurs—and distant areas like axons and synapses [53]. Newly made mitochondria must be transported anterogradely to sites of intense metabolic demand, including growth cones, axon terminals, nodes of Ranvier, and synaptic boutons. On the other hand, damaged or dysfunctional mitochondria are transported backward to the soma for degradation through mitophagy. This bidirectional transport system helps keep mitochondrial distribution and quality control in neurons (Figure 4) [53].

Mitochondrial transport is mainly dependent on microtubules and involves motor and adaptor proteins that attach mitochondria to the cytoskeleton [54]. Anterograde movement is mediated by ATP-dependent kinesin motors that carry mitochondria toward synaptic terminals, while retrograde movement relies on dynein motors that direct mitochondria from the distal axons back to the cell body.

Loss of GDAP1 severely disrupts mitochondrial transport in neurons, as shown by a notable decrease in the proportion of motile mitochondria [13]. Notably, the pool of bidirectionally mobile mitochondria is reduced, impairing both anterograde transport of healthy organelles to distant regions and retrograde movement of damaged mitochondria back to the soma for degradation (Figure 4). Furthermore, anterograde transport exhibits reduced velocity and shorter displacement, indicating problems with the motility machinery or its regulatory pathways. These changes lead to abnormal mitochondrial distribution along axons, which may cause energy deficits, synaptic problems, and ultimately, neuronal death. The disruption of mitochondrial transport in *GDAP1*-deficient neurons emphasizes the vital role of GDAP1 in coordinating mitochondrial dynamics and strongly suggests that its dysfunction contributes to the development of CMT disease.

### 4.2. Calcium Signaling

**Figure 5:**
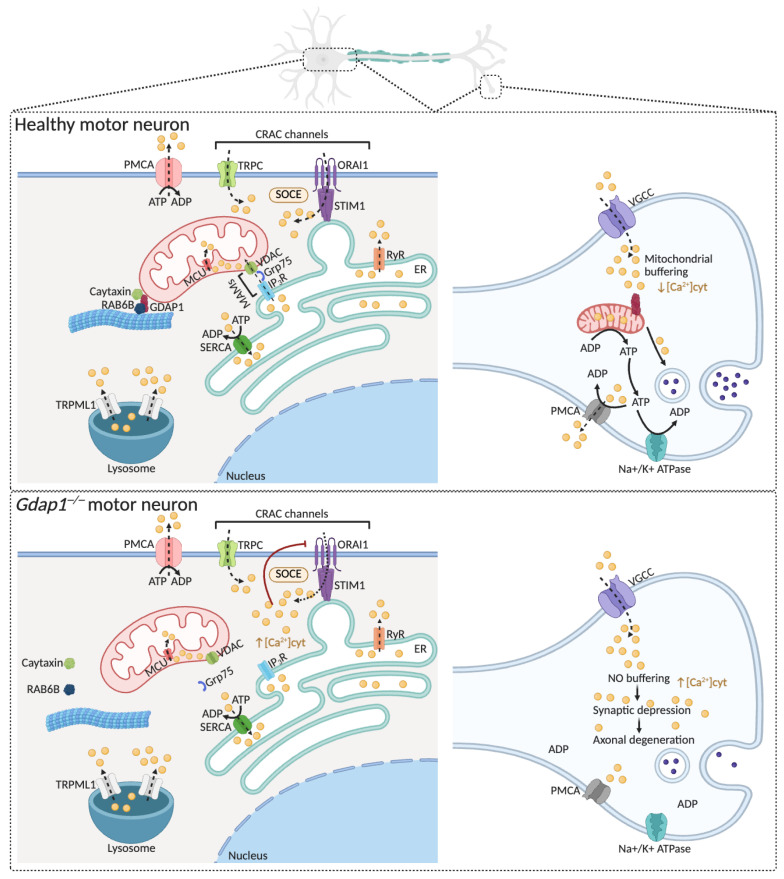
The lack of GDAP1 causes intracellular calcium-signaling defects. Upper panel: The ER acts as the main intracellular Ca^2+^ store, with Ca^2+^ uptake via SERCAs and release through RyRs and IP_3_Rs. Mitochondria quickly absorb released Ca^2+^ through VDAC and the MCU complex to boost metabolism. ER Ca^2+^ depletion activates STIM1 at ER–plasma membrane junctions, which triggers ORAI1-mediated SOCE. Additional Ca^2+^ influx occurs via TRPC channels, while PMCAs restore basal cytosolic Ca^2+^ levels. Lysosomes also serve as secondary Ca^2+^ stores, releasing Ca^2+^ through TRPML1 channels. GDAP1 interacts with RAB6 and Caytaxin to position mitochondria near ER–plasma membrane Ca^2+^ microdomains, promoting efficient Ca^2+^ signaling and neuronal health. At presynaptic terminals, VGCC-mediated Ca^2+^ entry is buffered by mitochondria to control neurotransmitter release. Lower panel: GDAP1 deficiency disrupts its interaction with RAB6B and Caytaxin, impairing proper mitochondrial placement at subplasmalemmal microdomains and altering SOCE activity. In synapses, defective mitochondrial transport reduces delivery of healthy mitochondria to distal regions, decreasing calcium buffering and leading to synaptic depression and axonal degeneration, ultimately impairing neuronal function. Abbreviations: ER: endoplasmic reticulum; Ca^2+^: calcium; ORAI1: calcium release-activated calcium channel protein 1; SERCA: sarcoplasmic reticulum Ca^2+^-ATPases; RyRs: ryanodine receptors; IP_3_R: inositol 1,4,5-trisphosphate receptors; VDAC: voltage-dependent anion channels; MCU: mitochondrial Ca^2+^ uniporter complex; STIM1: stromal interaction molecule 1; SOCE: store-operated Ca^2+^ entry; TRPCs: transient receptor potential channels; PMCA: plasma membrane Ca^2+^-ATPases; TRPML1: TRP mucolipin 1; RAB6: Ras-related protein Rab-6A; VGCCs: voltage-gated Ca^2+^ channels. Illustration created with BioRender. Lara Cantarero (2026). https://www.biorender.com/.

Calcium ions (Ca^2+^) serve as essential intracellular messengers that control many vital processes in eukaryotic cells, making calcium signaling both adaptable and widespread [55]. In neurons, Ca^2+^ is especially important for synaptic activity, energy production, and neurotransmission [56,57]. The spatial and temporal distribution of Ca^2+^ is finely regulated by a complex network of channels, transporters, and pumps located across the plasma membrane and organelles (Figure 5) [58]. Mitochondria are key regulators of intracellular Ca^2+^ dynamics and homeostasis, functioning both as buffers and modulators of calcium-dependent signaling pathways [59]. Due to its central role in neuronal function, disturbances in calcium homeostasis are linked to the development of many neurological disorders [60,61,62].

#### GDAP1 Deficiency Causes an Impaired Calcium Buffering and SOCE Inhibition, Inducing ER Stress

GDAP1 deficiency disrupts the store-operated calcium entry (SOCE) mechanism [15,63,64] (Figure 5). This mechanism begins when stromal interaction molecules STIM1 and STIM2 detect the depletion of ER calcium stores. Upon sensing calcium depletion, these proteins oligomerize and move to ER–plasma membrane junctions. At the same time, calcium channels formed by Orai1 and transient receptor potential cation (TRPC) family proteins cluster in opposing regions of the plasma membrane, facilitating calcium influx. Lysosomal TRP mucolipin-1 (TRPML1) channels also assist in endo-lysosomal Ca^2+^ release and membrane trafficking regulation. Effective SOCE depends on the strategic positioning of mitochondria near subplasmalemmal Ca^2+^ microdomains, where they regulate local calcium levels and prevent calcium-dependent channel inactivation. The plasma membrane Ca^2^ maintains cytosolic Ca^2+^ balance^+^-ATPase (PMCA) and the ER Ca^2+^-ATPase (SERCA), which remove Ca^2+^ from the cytosol by pumping it out or sequestering it into the ER lumen. The coordinated activity and dynamic crosstalk among Orai1, TRP channels, and Ca^2+^-ATPases integrate local and global calcium signaling, ensuring the accuracy of calcium-dependent signaling pathways and cellular responses to both physiological and pathological stimuli.

GDAP1 interacts with microtubules through β-tubulin, RAB6 and Caytaxin [15,64]. RAB6, a small GTPase associated with Golgi-derived vesicular trafficking, has been implicated in the recruitment of motor protein complexes to cargo along microtubules [65]. Caytaxin, a neuron-specific protein linked to microtubule dynamics, interacts with RAB6 and has been proposed to function as an adaptor connecting mitochondria to the microtubule-based transport machinery [66]. In this context, GDAP1 is thought to associate with RAB6–Caytaxin complexes, thereby facilitating the attachment of mitochondria to kinesin- and dynein-driven motors. These interactions are crucial for correct mitochondrial positioning and movement along the microtubule network. The loss of GDAP1 causes impaired mitochondrial trafficking and mislocalization away from subplasmalemmal calcium signaling domains [15,64]. This spatial disorganization hampers their ability to support SOCE, leading to decreased calcium influx and dysregulation of intracellular calcium balance. Additionally, clinical variants of *GDAP1* show distinct effects on SOCE activity [63]. Recessive *GDAP1* variants cause a reduction in SOCE activity, indicating a complete loss of function. Conversely, dominant *GDAP1* variants significantly increase SOCE activity, implying a gain of function. These results suggest that recessive and dominant variants operate through loss-of-function and gain-of-function mechanisms, respectively, likely by disrupting specific protein–protein interactions. Such interactions—possibly involving Caytaxin and RAB6B—may be essential for correctly positioning mitochondria near SOCE sites.

Cells lacking GDAP1 show cytosolic Ca^2+^ overload along with decreased mitochondrial Ca^2+^ levels (Figure 5) [13,15], a dysregulation that contributes to axonal spheroids formation and promotes axonal degeneration.

Moreover, the response of embryonic motor neurons (eMNs) to glutamate stimulation is delayed in the absence of GDAP1. Although *Gdap1^−^/^−^* eMNs show similar peak cytosolic Ca^2+^ transients in response to glutamate compared with wild-type cells, the half-time decay of cytosolic Ca^2+^ is significantly prolonged, and many neurons fail to restore basal Ca^2+^ levels [13]. This delay is not due to ER-mitochondria Ca^2+^ signaling, as NMDA-induced RyR-dependent Ca^2+^ release is similar in both genotypes. Rather, it reflects impaired mitochondrial positioning and dynamics, which compromise Ca^2+^ buffering at sites of high influx and energy demand. Coupled with the basal bioenergetic deficit in *Gdap1^−^/^−^* eMNs [13], these defects reduce the capacity of mitochondria to rapidly sequester Ca^2+^ and supply ATP for Na^+^/Ca^2+^ extrusion, slowing the neuronal response to glutamate, and suggesting a role of GDAP1 in maintaining calcium balance during synaptic activity.

*GDAP1*-deficient cells also show increased levels of eIF2α and ER chaperones like BiP and GRP94, indicating that a reduction in GDAP1 triggers ER stress as well [13].

### 4.3. Mitochondrial Bioenergetics and Antioxidant Defense

**Figure 6:**
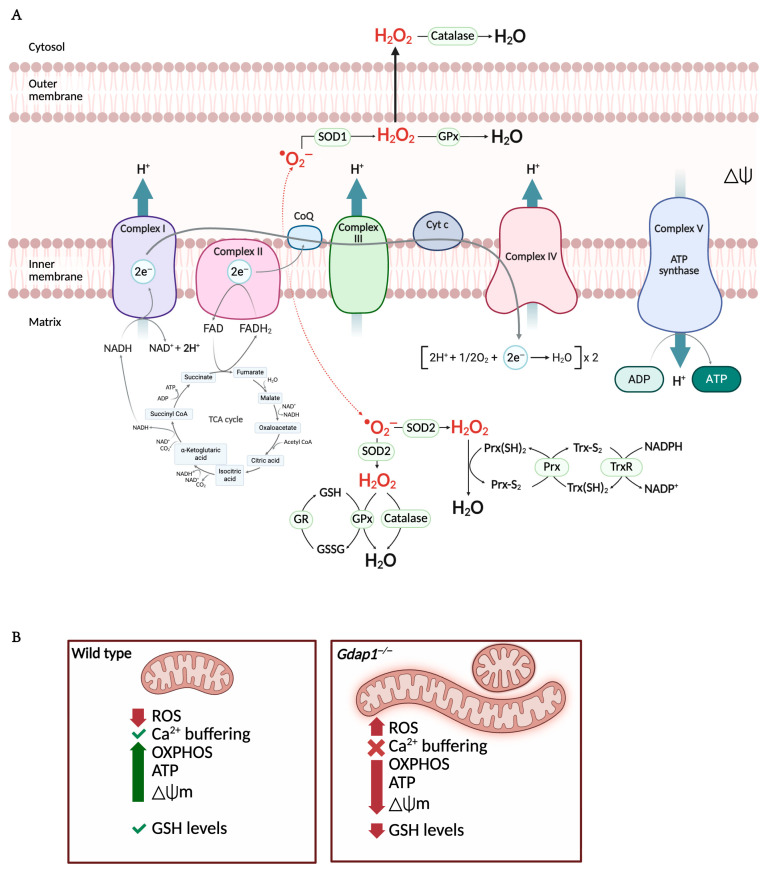
Mitochondrial oxidative phosphorylation and redox imbalance are altered in GDAP1-deficient cells. (**A**) The mitochondrial electron transport chain (ETC) comprises five protein complexes embedded in the inner mitochondrial membrane. The TCA cycle in the matrix provides NADH and FADH_2_ to fuel electron transfer, driving proton (H^+^) translocation into the intermembrane space. This generates the protonmotive force, or mitochondrial membrane potential (ΔΨ), which powers ATP synthesis via Complex V as protons flow back into the matrix. Reactive oxygen species (ROS) are generated at multiple ETC sites, primarily as superoxide (•O_2_^−^), which is converted to hydrogen peroxide (H_2_O_2_) by SOD1 and SOD2. Detoxification systems—including the glutathione peroxidase/reductase (GPx/GR) and peroxiredoxin/thioredoxin (Prx/TrxR) pathways, along with catalase—maintain redox balance within the matrix and cytosol. (**B**) In GDAP1 deficient cells, elongated and swollen mitochondria produce ROS, decreasing the OXPHOS, ATP production and the mitochondrial membrane potential. Moreover, the levels of cellular glutathione are also diminished, exacerbating oxidative stress. Abbreviations: ETC: electron transport chain; TCA: Tricarboxylic acid cycle; NADH: nicotinamide adenine dinucleotide (reduced); FADH_2_: flavin adenine dinucleotide (reduced); H^+^: proton; ATP: adenosine triphosphate; ROS: reactive oxygen species; •O_2_^−^: superoxide; H_2_O_2_: hydrogen peroxide; SOD1/2: Superoxide dismutase 1/2; GPx/GR: glutathione peroxidase/reductase; Prx/TrxR: peroxiredoxin/thioredoxin; OXPHOS: oxidative phosphorylation. Illustration created with BioRender. Lara Cantarero (2026). https://www.biorender.com/.

Mitochondria produce cellular energy in the form of adenosine triphosphate (ATP) through oxidative phosphorylation (OXPHOS) (Figure 6). This energy production, especially vital in neurons, is essential for axonal growth, maintaining membrane potential, and the release and reuptake of neurotransmitters at synaptic terminals [67].

#### Energetic Collapse in GDAP1 Deficiency: Role of Redox Homeostasis and Glutathione Metabolism

In cells lacking GDAP1, mitochondrial energy production is impaired. A consistent decrease in mitochondrial membrane potential (ΔΨm) is observed, along with reduced basal and ATP-linked respiration (oxygen consumption associated with ATP synthesis) [13]. Conversely, maximal uncoupled respiration, proton leak-related respiration, and non-mitochondrial oxygen consumption largely remain unaffected. These changes coincide with a significant drop in ATP generation, indicating disrupted mitochondrial energy function. It is suggested that problems in electron transport lead to ΔΨm dissipation and lower efficiency of oxidative phosphorylation (OXPHOS), resulting in an energetic failure (Figure 6) [13,68].

Evidence from *Gdap1*-deficient animal models [13,51,69], stable cell lines or iMNs [47,70], and fibroblasts from *GDAP1* patients [71] further demonstrates alterations in cellular oxidative stress, pointing to a critical role for GDAP1 in maintaining redox balance. Overexpression experiments show increased GSH levels and greater resistance to oxidative stress, while knockdown or disease-associated mutations make cells more susceptible to redox imbalance [12,71,72].

These findings imply that GDAP1 does not act as a typical GST enzyme but instead functions as a redox sensor, a function likely dependent on dimerization via cysteine 88 [25]. Reactive oxygen species can modulate proteins at redox-sensitive sites, including cysteine residues, through conformational changes such as disulfide-bond formation [73]. Dimerization of GDAP1 may therefore enable it to monitor the redox state of mitochondrial MCSs, not only with lysosomes but also with other organelles. Consistent with this model, both wild-type GDAP1 overexpression and redox modulation via GSH-MEE restore mitochondria–MCSs [12,74].

Overall, the current findings suggest that GDAP1 may indirectly modulate glutathione metabolism, possibly by regulating transporters or assembling redox-active complexes. Given the high oxidative load in long axons and peripheral nerves, this redox-regulatory role of GDAP1 is likely crucial to its neuroprotective function.

### 4.4. Neuroinflammation

#### GDAP1 Deficiency Triggers a Neuroinflammatory Process

**Figure 7:**
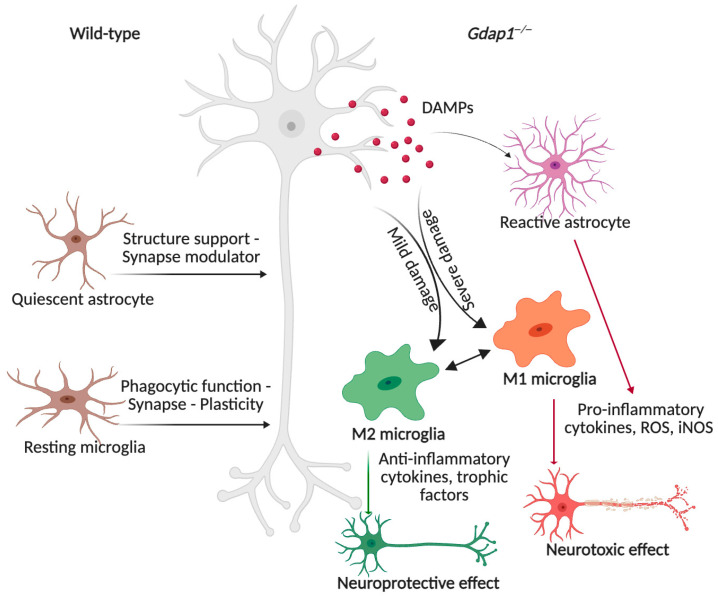
GDAP1 deficiency triggers a neuroinflammatory response. Under basal conditions, microglia are in a state of rest. Loss of GDAP1 leads to upregulation of inflammatory signaling pathways and pronounced reactive gliosis, with increased expression of astrocytic markers (GFAP, S100β) and the microglial marker IBA1. While M2 microglia levels remain unchanged, M1 microglia are markedly elevated, indicating a shift toward a proinflammatory, neurotoxic phenotype. Increased levels of TNF-α, pERK and complement components accompany this activation. Therefore, GDAP1 deficiency contributes to neurodegeneration through chronic glial activation and neuroinflammatory mechanisms. Illustration created with BioRender. Lara Cantarero (2026). https://www.biorender.com/.

Neuroinflammation has been extensively studied in demyelinating CMT models, but its role in axonopathy-associated CMT has only recently been recognized. The absence of Gdap1 correlates with the upregulation of inflammatory signaling pathways (Figure 7) [75]. In *Gdap1^−^/^−^* mice, there is pronounced reactive gliosis in the spinal cord, marked by increased expression of astrocytic markers GFAP and S100β, as well as the microglial marker IBA1. Normally, astrocytes provide neurotrophic effects by secreting neurotrophic factors like NGF and BDNF, along with S100. However, when they are persistently activated, they can adopt a pro-inflammatory phenotype, releasing cytokines, reactive oxygen species (ROS), and inducible nitric oxide synthase (iNOS), which can exacerbate neuronal damage [76,77,78].

In the *Gdap1^−^/^−^* mice, there is no change in M2 microglia (CD206+ve expression) typically associated with anti-inflammatory cytokines, debris clearance, and tissue repair. In contrast, there is a significant increase in M1 microglia (CD86+ve expression), which produce pro-inflammatory cytokines, ROS, and iNOS, contributing to neuronal injury and axonal damage (SMI-32+ve expression). This suggests that *GDAP1* deficiency promotes neurotoxic microglial activation and a detrimental inflammatory response in the spinal cord. Additionally, activated microglia signal to neighboring astrocytes, further amplifying neurotoxicity and tissue damage. This glial activation is accompanied by elevated levels of inflammatory mediators, including TNF-α, phosphorylated ERK, and complement components C1qa and C1qb [75]. Notably, these inflammatory alterations coincide with abnormal synaptic molecular signatures, suggesting that chronic glial activation and neuroinflammatory mechanisms may contribute to *GDAP1* deficiency pathophysiology and neurodegeneration.

### 4.5. GDAP1 as a Hub for Mediating Interactions Between Mitochondria and Organelles

**Figure 8:**
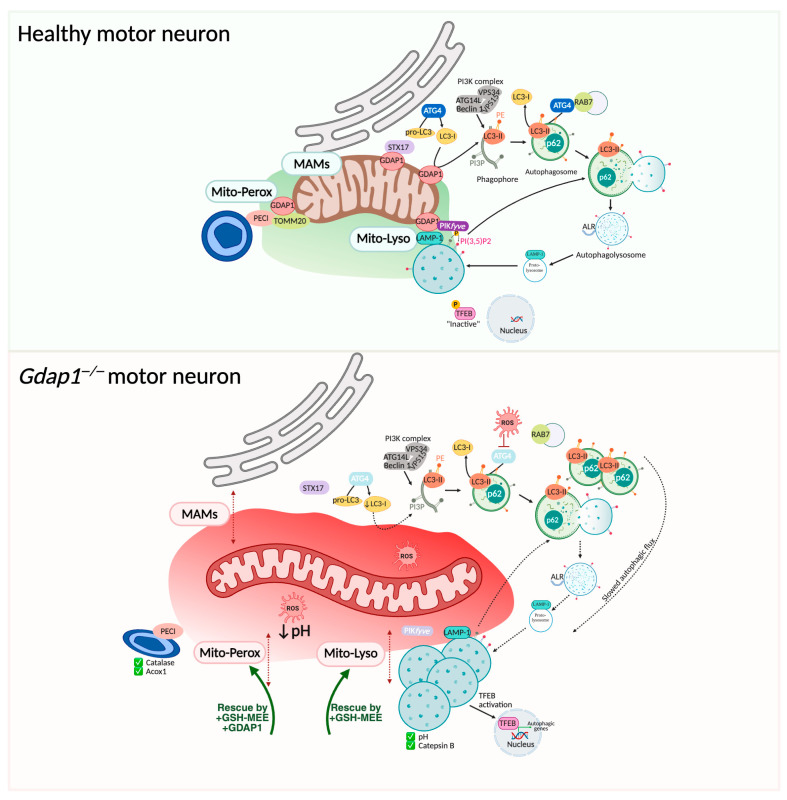
GDAP1 forms part of mitochondrial membrane contact sites. Upper panel: GDAP1 is in the OMM regulating mitochondrial MCSs. GDAP1 participates in membrane biogenesis of early autophagic vesicles, facilitating proper basal autophagic flux; and in later stages, interacting with the kinase PIK*fyve*. Lower panel: GDAP1 deficiency disrupts mitochondrial MCSs, increasing the distance between mitochondria, the ER, lysosomes, and peroxisomes. This disorganization is associated with altered redox conditions, impaired vesicle formation at MAMs, reduced autophagic flux, and enlarged lysosomes. Illustration created with BioRender. Lara Cantarero (2026). https://www.biorender.com/.

The OMM serves as a key platform for forming membrane contact sites (MCSs) with various organelles, enabling the direct transfer of ions, lipids, and metabolites. Among these, mitochondria-associated membranes (MAMs) formed with the ER are the most well-studied. In addition to the ER, the OMM creates functional contacts with the plasma membrane, peroxisomes, and lysosomes, positioning mitochondria as central hub in cellular signaling networks (Figure 8) [8]. Disruption of these communication centers between organelles is increasingly linked to the development of neurological disorders [10]. The importance of mitochondria–organelle interactions is further underscored by the fact that mutations in mitochondrial proteins often lead to structural and functional changes in lysosomes and peroxisomes. Additionally, many peroxisomal and lysosomal disorders show secondary mitochondrial disturbances [79]. In this section, we discuss the role of GDAP1 in mitochondrial–MCSs functioning.

#### 4.5.1. Mitochondria-Associated Membranes and Basal Autophagy

MAMs are specialized subdomains of the ER that form reversible contacts with mitochondria. These ER–mitochondria contacts serve as key sites for the regulation of calcium transport, phospholipid metabolism, autophagy initiation, mitochondrial dynamics, and cellular survival [80,81]. MAMs are described as the initial platforms for phagophore formation in basal autophagy [82]. Efficient autophagy depends on proper membrane trafficking, remodeling, and precise organelle positioning and dynamics at multiple steps of the pathway [83,84]. In fact, dysregulation of autophagy is a common feature in neurodegenerative diseases, where impaired autophagic flux contributes to the accumulation of damaged proteins and organelles, exacerbating neuronal stress and degeneration [85,86].

GDAP1 is localized at MAMs [12,15], and its deficiency reduces both the frequency and proximity of these contacts [13]. Additionally, the accumulation of autophagic vesicles has been reported in GDAP1-deficient cells, implicating disrupted basal autophagy in the pathophysiology of GDAP1-related CMT (Figure 8) [12,49]. During autophagy initiation, the Q-SNARE protein syntaxin 17 (STX17) recruits ATG14 to MAMs, facilitating autophagosome formation [87]. GDAP1 constitutively interact with STX17, suggesting a role in recruiting additional factors to MAMs to coordinate autophagosome biogenesis. The elongation of the phagophore and autophagosome closure depend on LC3 lipidation, where LC3 is covalently attached to phosphatidylethanolamine (PE) through ubiquitin-like enzymatic reactions. Mitochondria, as the main PE source, and MAMs, as sites of lipid synthesis and trafficking, are thus essential for this process [88]. GDAP1 interacts with LC3-I under basal conditions and with LC3-I/II when autophagy is induced or inhibited. Both LC3-I and LC3-II are found in ER and MAM fractions, supporting MAM-associated lipidation [12]. Notably, GDAP1 deficiency causes a significant reduction of LC3-I and LC3-II levels in MAM fractions, suggesting impaired lipidation at this critical site.

In later stages of autophagy, GDAP1 interacts with the lysosomal kinase PIK*fyve*, which phosphorylates PI3P into PI(3,5)P2 to regulate vesicle maturation, identity, and trafficking [89,90]. This interaction increases under starvation-induced autophagy. When GDAP1 is depleted, cells show enlarged, perinuclear lysosomes with abnormal distribution, a phenotype that suggests decreased PIK*fyve* activity. Pharmacologically inhibiting PIK*fyve* in control cells produces a similar lysosomal phenotype, confirming that GDAP1 is essential for proper PIK*fyve* function. Loss of GDAP1 also causes increased nuclear translocation of transcription factor EB (TFEB), implying impaired lysosomal regulation and autophagy. Although lysosomal pH and cathepsin B activity remained normal, morphological changes after Bafilomycin A1 treatment point to defects in autophagic lysosome reformation (ALR), a cellular process responsible for regenerating functional lysosomes following autophagy, further supporting the role of GDAP1 in PIK*fyve*-mediated lysosomal maturation.

Functionally, GDAP1 loss strongly induces a disruption of both early and late stages of autophagy, leading to altered phagophore and autophagosome morphology, accumulation of autophagosomes, impaired vesicle maturation, slowed autophagic flux, and defective vesicle–lysosome fusion [12]. These findings indicate that GDAP1 is essential for coordinating autophagosome formation, maturation, and trafficking, linking mitochondrial function and MAMs integrity to effective cellular quality control mechanisms. Impaired autophagy likely contributes to the neurodegenerative phenotype observed in GDAP1-related CMT.

#### 4.5.2. Mitochondria–Lysosomes Contact Sites

While ER-mitochondria contacts have been recognized for years, recent research has expanded this understanding to include interactions with other organelles including mitochondria–lysosomes contacts [91,92,93]. These contacts allow bidirectional regulation of mitochondrial and lysosomal dynamics. They are facilitated by active GTP-bound lysosomal Ras-related protein Rab-7 (RAB7), and contact untethering was mediated by the recruitment of TBC1 Domain Family Member 15 (TBC1D15) to mitochondria via FIS1. Functionally, lysosomal contacts mark sites of mitochondrial fission, allowing lysosomes to regulate mitochondrial networks, whereas mitochondrial contacts regulate lysosomal RAB7 hydrolysis through TBC1D15 [93]. Recently, a novel signaling mechanism called NiMA (Nutrient-induced Mitochondrial Activity) has been described, which is blocked by amyloid-β oligomers in the Alzheimer’s disease brain [94]. NiMA coordinates the functional interplay between mitochondria and lysosomes in response to nutrient availability, regulating mitochondrial fission, bioenergetics, and lysosomal activity to optimize cellular metabolism [94]. Through this bidirectional communication, lysosomes can influence mitochondrial network remodeling, while mitochondria modulate lysosomal enzyme activity and trafficking. This interdependence may explain the coordinated alterations observed in both organelles across various human diseases, including neurodegenerative disorders.

GDAP1 interacts with the lysosomal protein LAMP1 to regulate mitochondria–lysosome MCSs [12,37,52]. In cells lacking GDAP1, these contacts are reduced, leading to altered lysosomal morphology—manifested as enlarged and aggregated lysosomes—without affecting their enzymatic activity (Figure 8). Interestingly, clinical variants of GDAP1 have different effects on mitochondria–lysosome contacts [52]: recessive variants decrease these MCSs, while dominant variants cause a significant increase. Remarkably, restoration of cellular glutathione levels with GSH-MEE supplementation rescues these defects, highlighting GDAP1’s essential role in maintaining the redox environment needed for MCS stability. GDAP1 deficiency also lowers the pH in these mitochondria–lysosome contact sites, further emphasizing the importance of a finely tuned redox microenvironment in the regulation of inter-organelle communication [74].

#### 4.5.3. Mitochondria–Peroxisomes Contact Sites

These MCSs have also been described and exhibit a close functional interplay that impacts human health and development. The couple of tethering proteins in these MCSs are Fzo1 (mitofusins 1 and 2 yeast orthologue) in mitochondria and peroxin 19 (Pex19) or peroxin 14 (Pex14) in peroxisomes [95]. The metabolic cooperation of peroxisomes and mitochondria in the β-oxidation of fatty acids and detoxification of ROS are the best-studied examples of this cross talk [96]. Remarkably, peroxisomes and mitochondria share key proteins of their division machinery (DRP1, Mff, and Fis1), which suggests coordinated division under certain conditions. Moreover, mitochondria have an essential role in de novo peroxisome biogenesis [97]. Several peroxisomal disorders show mitochondrial defects [96], and peroxisomal dysfunction impairs the function of peripheral nerves, affecting Ranvier node structures [98], where inter-organelle contacts are essential.

GDAP1 also plays a crucial role in peroxisomal biology [74,99]. Its deficiency disrupts both the number and morphology of peroxisomes-including those within motor neuron axons-without impacting their enzymatic capacity (Figure 8) [74]. This change reflects the cellular phenotype seen in lysosomal dynamics. Additionally, GDAP1-deficient cells show a significant reduction in mitochondria–peroxisome contacts, a defect that can be corrected either by glutathione supplementation or by overexpressing GDAP1.

**Figure 9:**
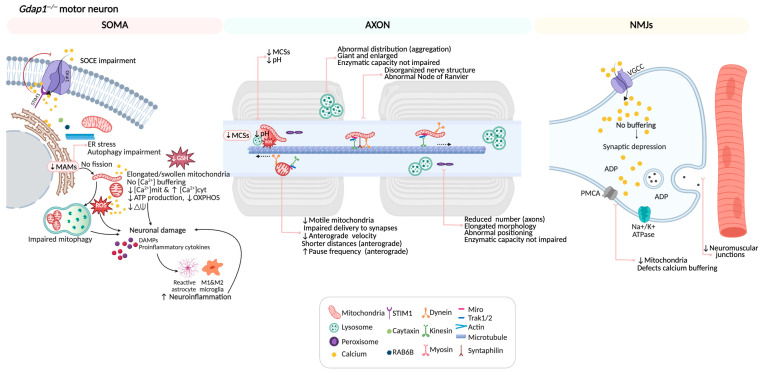
Summary of pathological cellular phenotypes in a GDAP1-deficient motor neuron. GDAP1 is a multifunctional protein that regulates key processes essential for neuronal integrity. It modulates mitochondrial dynamics by balancing fission and fusion events, maintains proper mitochondrial distribution, and contributes to the formation and regulation of mitochondria MCSs with the ER, lysosomes, and peroxisomes. Through these interactions, GDAP1 participates in calcium signaling and autophagic vesicles biogenesis. Additionally, GDAP1 supports redox homeostasis by limiting reactive oxygen species (ROS) accumulation and preserving mitochondrial membrane potential and ATP production. When GDAP1 is absent, these interconnected pathways fail leading to altered Ca^2+^ handling, oxidative stress, defective autophagy, and ultimately neuronal dysfunction and degeneration. Illustration created with BioRender. Lara Cantarero (2026). https://www.biorender.com/.

Disruption of mitochondrial MCSs causes axonal structural issues [74], emphasizing the vital role of GDAP1 in maintaining axonal health. Together, these results identify GDAP1 as a multifunctional protein that manages inter-organelle interactions, calcium homeostasis, redox signaling, and axonal stability (Figure 9).

## 5. Therapeutic Perspectives and Future Directions in CMT-*GDAP1*

**Figure 10:**
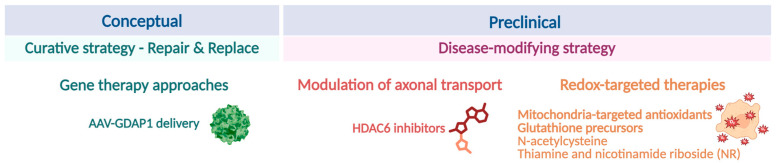
Therapeutic perspectives and future directions in CMT-GDAP1. Proposed interventions include gene replacement therapy, modulation of microtubule-dependent axonal transport, and redox-targeted therapies aimed at reducing oxidative stress and mitigating downstream cellular dysfunction. These approaches differ in their level of maturity and translational potential. Illustration created with BioRender. Lara Cantarero (2026). https://www.biorender.com/.

Elucidating the molecular and cellular functions of GDAP1 in mitochondrial interactions and biology not only deepens our understanding of Charcot–Marie–Tooth disease pathophysiology but also uncovers new therapeutic possibilities. The unique position of GDAP1 at the interface of mitochondria interactions with endoplasmic reticulum, lysosomes, and peroxisomes suggests that this protein is a crucial regulator of organelle communication, calcium buffering, and redox balance. Therapeutic strategies that aim to restore GDAP1 function, compensate for its absence, or target its downstream pathways are promising options, not only for CMT but also for other neurodegenerative diseases and peripheral neuropathies that share several common pathophysiological mechanisms involving the mitochondria. Considering the broad spectrum of cellular problems caused by GDAP1 deficiency, a combination of molecular, genetic, and pharmacological approaches may be required. Below, we hierarchize these approaches according to their level of maturity and translational potential (Figure 10).

### 5.1. Conceptual, Curative Strategy

#### Gene Therapy Approaches

Gene replacement therapy is one of the most promising strategies for CMT neuropathies [100]. Adeno-associated virus (AAV)-mediated delivery of wild-type *GDAP1* can potentially restore protein levels and re-establish inter-organelle contacts, thereby correcting bioenergetic and signaling deficits. Promising results from related inherited peripheral neuropathies provide strong translational justification for advancing this approach in clinical settings.

Despite this promise, several challenges remain, including efficient delivery to peripheral neurons, long-term expression, neuronal specificity, and the optimal therapeutic window [100,101]. In particular, the timing of intervention may be critical, as advanced axonal degeneration may limit the reversibility of the disease phenotype. These considerations highlight the need for further preclinical studies to evaluate efficacy, safety, and optimal delivery strategies.

### 5.2. Preclinical, Disease-Modifying Strategy

#### 5.2.1. Modulation of Axonal Transport

Given that GDAP1 participates in mitochondrial axonal transport, small molecules that stabilize microtubule-dependent transport may restore organelle distribution and axonal integrity. Most therapy efforts for CMT have been focused on this final common pathway of axonal degeneration, which occurs as a primary mechanism in axonal CMT and as a secondary consequence in demyelinating forms [102].

HDAC inhibitors have become potential candidates for treating neuropathies. Currently, five FDA-approved HDAC inhibitors are on the market. Notably, many efforts have focused on developing selective HDAC6 inhibitors. HDAC6 plays a negative, deacetylase-dependent role in axonal transport and regeneration, both processes involved in axonal CMT [103]. The current insights into these HDAC6 functions in axonal CMT, along with its selective druggability targeting its deacetylase activity, make targeting HDAC6 particularly attractive. Since acetylation of α-tubulin is reduced in neurons from Gdap1-deficient mice [49], this therapeutic approach could also be applied to CMT-GDAP1 disease.

Nevertheless, this approach remains at preclinical stage [104], and its therapeutic efficacy is expected to be indirect and potentially limited to symptomatic improvement rather than correction of the primary defect. Moreover, long-term safety and specificity in peripheral neurons need to be carefully evaluated.

#### 5.2.2. Redox-Targeted Therapies

Redox-targeted therapies represent a more conceptual and supportive strategy aimed at mitigating downstream consequences of GDAP1 dysfunction. Given the proposed role of GDAP1 as a redox sensor, antioxidant-based approaches—such as mitochondria-targeted antioxidants (e.g., MitoQ) [105], glutathione precursors, N-acetylcysteine, thiamine and nicotinamide riboside (NR) [106], or small molecules enhancing endogenous antioxidant defenses—may mitigate oxidative stress and partially normalize organelle morphology and neuronal function.

While these interventions may provide neuroprotective benefits, their effects are likely to be modulatory. Moreover, antioxidant therapies do not address the underlying genetic defect and may show variable efficacy depending on disease stage, dosage, and cellular context. As such, they are best viewed as adjunctive strategies that could complement gene-based or pathway-targeted interventions.

## 6. Conclusions

Research on the basic biology of GDAP1 and its clinical variants suggests that this OMM protein acts as a central connector, linking interactions between mitochondria and other organelles. The dynamic nature of mitochondria allows them to coordinate signaling, molecular transport, and structural attachment, helping to keep the cell balanced. This adaptability enables mitochondria to quickly respond to changes in energy needs and stress, highlighting the critical role of the OMM and its proteins in cell resilience.

GDAP1 exemplifies the consequences of OMM impairment, as pathogenic variants of this protein disrupt mitochondrial dynamics, calcium buffering, redox signaling, and communication between organelles. This dysfunction is especially noticeable in neurological disorders, emphasizing how problems at the mitochondrial interface spread cellular damage. Understanding the role of GDAP1 helps clarify the mechanistic links between mitochondrial membrane integrity and the development of neurodegenerative diseases. Given the role of GDAP1 in the complex crosstalk between mitochondria and other organelles, therapeutic approaches that restore cellular homeostasis and the associated nerve pathophysiology of GDAP1 deficiency may offer effective treatments for common diseases rooted in mitochondrial dysfunction. This integrative perspective is essential for the development of comprehensive therapies in neurodegenerative and peripheral neuropathies.

## Data Availability

No new data were created or analyzed in this study.
